# Environmental niche and flight intensity are associated with molecular evolutionary rates in a large avian radiation

**DOI:** 10.1186/s12862-022-02047-0

**Published:** 2022-08-02

**Authors:** Paola Montoya, Carlos Daniel Cadena, Santiago Claramunt, David Alejandro Duchêne

**Affiliations:** 1 Instituto de Investigación de Recursos Biológicos Alexander von Humboldt, Avenida Circunvalar # 16-20, Bogotá, Colombia; 2 Departamento de Ciencias Biológicas, Universidad de los Andes, Apartado, 4976 Bogotá, Colombia; 3 Department of Natural History, Royal Ontario Museum, 100 Queen’s Park Crescent, Toronto, ON M5S 2C6 Canada; 4 Department of Ecology and Evolutionary Biology, University of Toronto, Toronto, ON M5S 1A1 Canada; 5 Centre for Evolutionary Hologenomics, University of Copenhagen, Øster Farimagsgade 5A, 1352 Copenhagen, Denmark

**Keywords:** Furnariidae, Flight ability, Hand-wing index, Environmental temperature, Environmental UV radiation

## Abstract

**Background:**

Metabolic activity and environmental energy are two of the most studied putative drivers of molecular evolutionary rates. Their extensive study, however, has resulted in mixed results and has rarely included the exploration of interactions among various factors impacting molecular evolutionary rates across large clades. Taking the diverse avian family Furnariidae as a case study, we examined the association between several estimates of molecular evolutionary rates with proxies of metabolic demands imposed by flight (wing loading and wing shape) and proxies of environmental energy across the geographic ranges of species (temperature and UV radiation).

**Results:**

We found weak evidence of a positive effect of environmental and morphological variables on mitochondrial substitution rates. Additionally, we found that temperature and UV radiation interact to explain molecular rates at nucleotide sites affected by selection and population size (non-synonymous substitutions), contrary to the expectation of their impact on sites associated with mutation rates (synonymous substitutions). We also found a negative interaction between wing shape (as described by the hand-wing index) and body mass explaining mitochondrial molecular rates, suggesting molecular signatures of positive selection or reduced population sizes in small-bodied species with greater flight activity.

**Conclusions:**

Our results suggest that the demands of flight and environmental energy pose multiple evolutionary pressures on the genome either by driving mutation rates or via their association with natural selection or population size. Data from whole genomes and detailed physiology across taxa will bring a more complete picture of the impact of metabolism, population size, and the environment on avian genome evolution.

**Supplementary Information:**

The online version contains supplementary material available at 10.1186/s12862-022-02047-0.

## Background

The factors driving molecular evolution are a long-standing matter of interest in the biological sciences. Several life-history traits [1], metabolic activity [2], and environmental energy [3] correlate with molecular evolutionary rates across multiples clades. These factors are also likely to interact, with the evolutionary outcome depending on the balance between the costs of sources of deleterious mutations and the requirements of the particular lifestyle of species [4]. Several studies have shown interactions among multiple correlates of molecular evolution [5, 6], but there is still a limited understanding of the relative contribution of biological and environmental factors to molecular rates.

Metabolic rate is an important putative driver of molecular evolutionary rates because of the mutagenic effects of metabolism [7, 8]. Specifically, metabolism produces oxygen and nitrogen free-radicals which may cause mutations through damage to DNA [9]. Mass-specific metabolic rate is inversely associated with body mass [10], which provides one explanation for the accelerated rates of molecular evolution in small vertebrates compared to large ones. Although there is some evidence that metabolic rates are indeed associated with rates of molecular evolution [2, 7, 11], multiple studies have not found such an association [12–14].

An explanation for the mixed evidence of the impact of metabolism on molecular evolution is that there has been a heavy focus on body size and temperature as proxies of metabolic rate [14, 15], yet multiple biological factors can impact metabolic requirements. For example, flight in birds is an extreme form of endurance exercise associated with high metabolic rates [16, 17], high respiratory rates [12], high rates of catabolism of lipids and proteins [18], and oxidative stress [19]. An increase in flight demand in many bird species is likely to lead to higher and longer term exposure to reactive forms of oxygen and nitrogen, which have a mutagenic effect resulting from oxidative damage [20], and may generate faster rates of molecular evolution beyond the effects of temperature and body mass.

Alternatively, molecular evolution might not be dominated by mutagens driving the rate of mutation, but rather by the mutations enabling adaptations to highly energetically demanding lifestyles. Among the many adaptations in avian lineages for sustaining long periods of flight with high efficiency are a high capillary density in flight muscles, low wing loading, high wing aspect-ratio, and major changes in skeletal structures and tissues [21]. Flight demand might also lead to adaptations for lifestyles with high metabolic turnover (i.e., a ‘fast pace of life’): birds that undergo high flight demand are likely adapted to having rapid growth, high fecundity, and short lifespans [22] (but see [23]). The signatures for changes in molecular evolutionary rates as driven by flight demand might therefore be predominant in regions of the genome influenced primarily by selection, rather than mutation. In the absence of whole-genome sequences for large numbers of closely related species, examining the relative contribution of synonymous and non-synonymous substitutions in coding regions offers a window into genomic regions under different selective regimes [24].

Another widely studied factor that might drive molecular evolutionary rates is environmental energy, through its mutagenic effect on the genome [8, 25]. Studies in organisms including plants [3], marine fishes [26], and lizards [2] have found associations between environmental energy and molecular rates. The link between energy and molecular evolution is often inferred using environmental temperature as a proxy of available energy [3]. Other studies have focused on UV radiation, a well understood mutagen [11, 27, 28]. Temperature and UV may also interact, such that DNA repair is less effective at high UV radiation but low temperatures [29]. However, it is unclear how UV and its interaction with temperature might impact the germline in many organisms, like endothermic vertebrates [30].

Environmental energy might also affect molecular evolution by mediating the fate of novel mutations, as opposed to affecting basal mutation rates. For example, temperature might influence the fitness effects of mutations, with maximal fitness occurring at around global maximal temperatures [31]. Greater energy availability might also facilitate the accumulation of biomass and allow high-energy environments to sustain larger numbers of individuals [32]; in larger populations, selection is more efficient and novel beneficial mutations are more likely to reach fixation rapidly [24, 33]. High energy availability might also lead to reduced investment in thermoregulation, allowing limited resources to be used in other activities (e.g. reproduction) and ultimately permitting novel adaptations [34].

We examined the association between the rates of molecular evolution and the metabolic demands imposed by flight and environmental energy across 230 species of the avian family Furnariidae, a large radiation of 294 passerine birds including the Neotropical ovenbirds and woodcreepers [35]. Furnariids have undergone fast and constant diversification over the last 30My [36, 37], and inhabit a broad range of habitats in South and Central America [38]. The species richness of the family and range of habitats it occupies across broad gradients of elevation, latitude, and environmental conditions [38] make it an uniquely diverse clade for testing the impact of physiology and environmental niche on molecular evolution.

Using data for the majority of species of Furnariidae (80%) we provide the first near-complete species-level examination of the correlates of molecular evolution in a large vertebrate radiation. We focused on measurements of the flight apparatus, and environmental temperature and UV radiation as proxies of metabolic demand and environmental energy, respectively. Using estimates of synonymous (*d*_S_) and non-synonymous substitution rates (*d*_N_), we assessed whether wing morphology and environmental energy are associated with molecular evolutionary rates in a set of nuclear and mitochondrial genomic regions. Following molecular evolutionary theory, individual tests using data from synonymous and non-synonymous gene sites allowed us to dissect the separate molecular signatures of selection or population size and mutation rate [39, 40]. Our findings suggest a nuanced picture of the impact of flight demands and environmental energy on genome evolution, and highlight several questions to be addressed in genome-level studies.

## Results

**Fig. 1:**
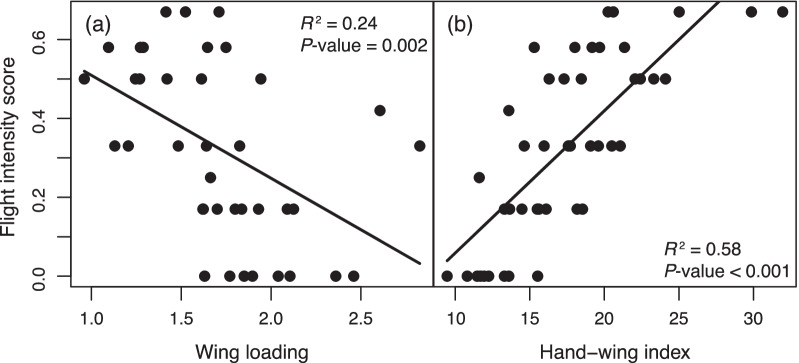
Association between a score of flight-intensive habits (0 = limited flight habits, 1 = intensive flight habits; see "Methods" section) with (**a**) wing loading and (**b**) hand-wing index across 44 genera of the avian family Furnariidae. Lines show fitted PGLS regression models

Wing morphology is related to the flight habits of species; for instance, species that fly more frequently tend to have more elongated wings (high aspect-ratio). We first examined this assumption in the Furnariidae by calculating a flight-habit score from a detailed exploration of the literature for the portion of species with well-known flight habits (see "Methods"; Additional file 1: Table S1). We found that the use of flight in Furnariidae as described by our flight-habit score was negatively associated with wing loading (*p* = 0.002, *R*^2^ = 0.24, Fig. 1) and positively associated with the wing’s aspect ratio as estimated by the hand-wing index (*p* < 0.001, *R*^2^ = 0.58; Fig. 1; full results available online, github.com/duchene/furnariidae_rates). Therefore, species with low wing loading and high aspect ratio (high hand-wing index) tend to fly more throughout their lives. Because flight habit is only understood in detail for a small number of species, we used wing morphology for further analyses assuming that it describes the metabolic demands associated with flight.

**Fig. 2:**
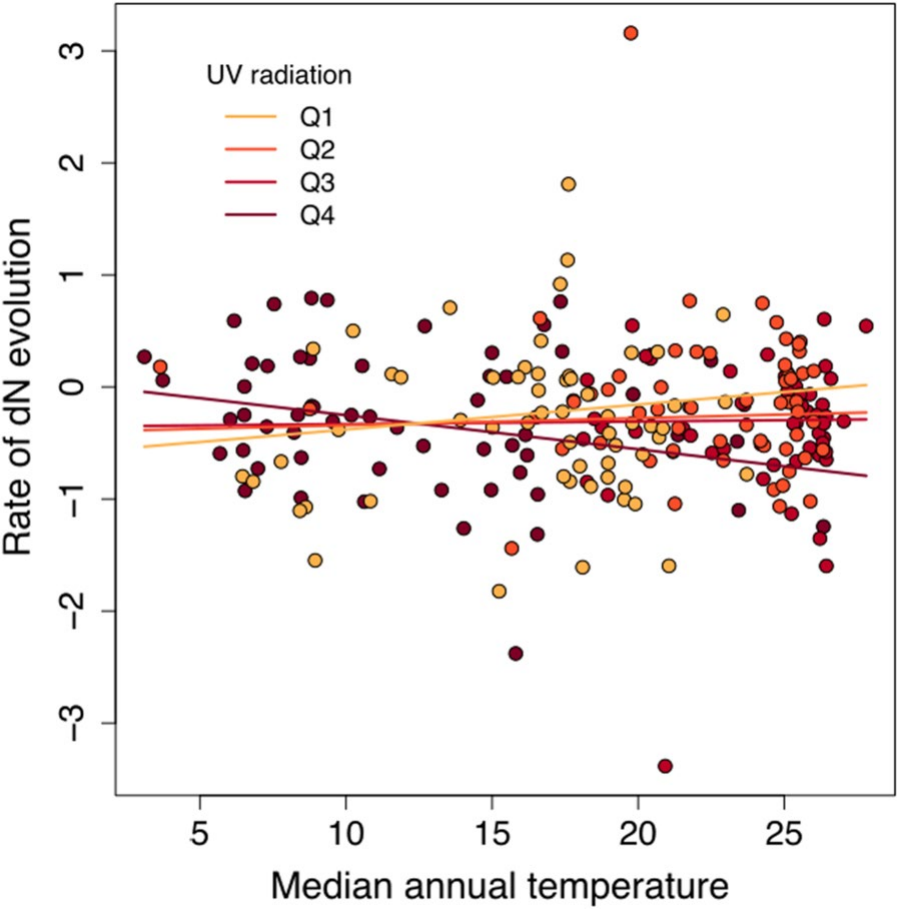
Association between temperature and mitochondrial *d*_N_ molecular rates across species of the avian family Furnariidae at different intensities of UV radiation. At high UV intensities molecular rates have a negative association with environmental temperature, while at low UV intensities the association between molecular rates and temperature is positive. Colours represent mid-quartile values of UV radiation. Darker colours indicate greater values. The predicted regression lines are also coloured for each of the mid-quartile values of UV radiation. Molecular *d*_N_ rates are shown normalized and under a Box-Cox transformation

We ran phylogenetic regressions exploring whether molecular evolutionary rates are predicted by wing morphology and environmental variables while considering the influence of body mass due to its known effect on metabolic rates (see "Methods"). Our models showed a weak positive effect of temperature and UV radiation on mitochondrial *d*_*N*_ substitution rates (Tables 1, 2; full models result available online, github.com/duchene/furnariidae_rates). They also showed moderate evidence of a negative interaction between the two environmental-energy variables in explaining mitochondrial *d*_N_ molecular rates whereby species of Furnariidae exposed to low temperatures and high UV radiation have faster rates of mitochondrial *d*_N_ (Table 1; Fig. 2).

**Fig. 3:**
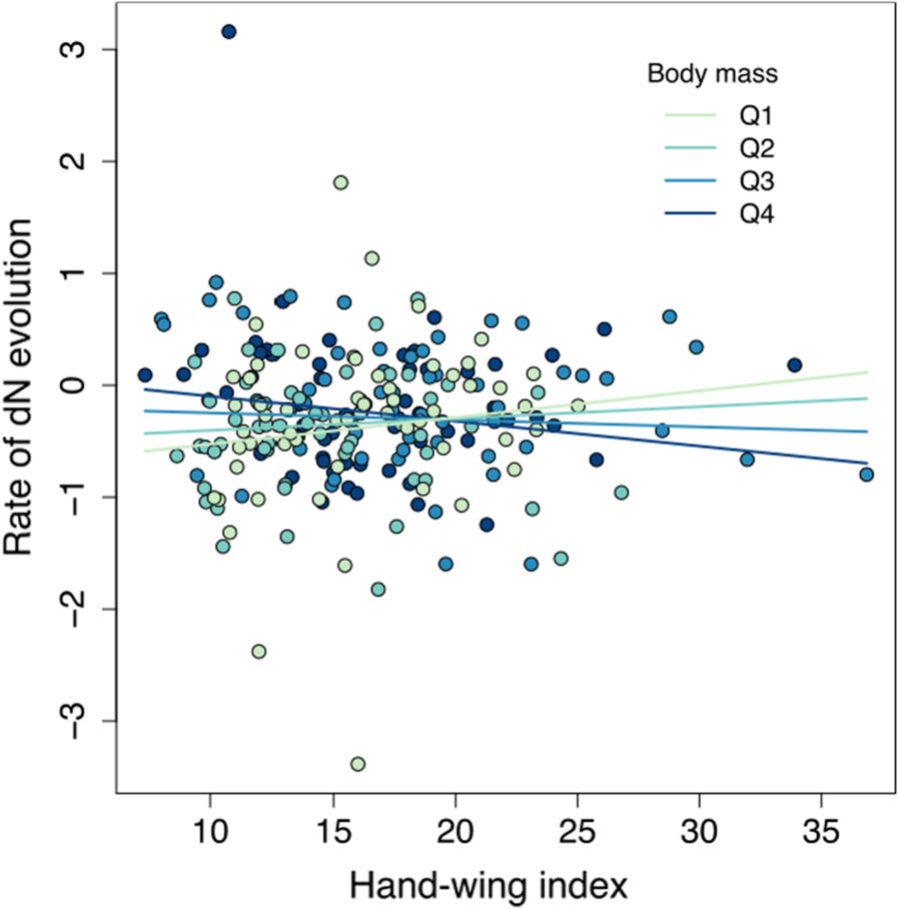
Association between the hand-wing index and mitochondrial *d*_N_ molecular rates across species of the avian family Furnariidae. The relationship changes with body mass, showing a positive association between molecular rates and hand-wing index in small-bodied species, whereas the association appears negative in large-bodied species. Colours represent mid-quartile values of body mass, where darker colours indicate species with greater body mass. The predicted regression lines are also coloured for each of the mid-quartile values of body mass. Molecular rates are shown normalized and under a Box-Cox transformation

The hand-wing index and body size showed a positive overall effect on mitochondrial molecular rates. Our models revealed weak evidence for an effect of hand wing-index on *d*_*N*_ and* d*_*N*_*/d*_*S*_ substitution rates, whereas body size showed a weak effect on *d*_*S*_ and *d*_*N*_*/d*_*S*_ molecular rates, and a moderate effect on *d*_*N*_ substitution rates (Table 1). In addition, the analyses indicated a weak negative interaction between hand-wing index and body size driving mitochondrial *d*_*N*_ and *d*_*N*_*/d*_*S*_ substitution rates (Fig. 3; Table 1). This interaction suggests a positive association between the hand-wing index and molecular rates in small-bodied species changing to a negative relationship in large-bodied species (Fig. 3).

Sensitivity tests excluding 25% of the species (see "Methods") showed that *t-*statistics distributions for the parameters estimated did not overlap with zero across randomisations (Additional file 1: Fig. S1–S2). These results suggest that the signal found with the complete data set does not seem to be driven by a subset of highly influential species.

**Table 1:** PGLS regression coefficients from models testing whether the hand-wing index and environmental variables explain molecular rates. A Box-Cox transformation was used for all molecular rates variables

Molecular rate response regression terms	Hand-wing index	Log body mass	Median annual temperature	Median UV radiation	Hand-wing index * log body mass	Median annual temperature * median UV radiation	*R* ^2^
Mitochondrial *d*_N_/*d*_S_	0.091*	0.442*	0.040	1.07E−04	− 0.026*	− 7.40E−06	0.023
Mitochondrial *d*_N_	0.115*	0.669**	0.079*	1.90E−04**	− 0.035*	− 1.51E−05**	0.047
Mitochondrial *d*_S_	0.091	0.617*	0.062	1.33E−04	− 0.025	− 1.12E−05	0.031
Nuclear *d*_N_/*d*_S_	0.081	0.471	− 0.037	− 1.57E−04	− 0.020	5.79E−06	0.045
Nuclear *d*_N_	0.033	− 0.024	0.070	1.39E−04	− 0.003	− 1.25E−05	0.020
Nuclear *d*_S_	−0.049	− 0.690	0.140	4.21E−04	0.020	− 2.58E−05	0.087

*P*-values denoted * ≤ 0.1. ** ≤ 0.05

**Table 2:** PGLS regression coefficients from models testing whether wing loading and environmental variables explain molecular rates. A Box-Cox transformation was used for all molecular rates variables

Molecular rate response regression terms	Wing loading	Log body mass	Median annual temperature	Median UV radiation	Wing loading * log body mass	Median annual temperature * median UV radiation	*R* ^2^
Mitochondrial *d*_N_/*d*_S_	0.318	0.274	0.042	1.00E−04	− 0.118	− 7.69E−06	0.015
Mitochondrial *d*_N_	0.149	0.093	0.084*	1.94E−04*	− 0.016	− 1.56E−05*	0.033
Mitochondrial *d*_S_	− 0.242	− 0.251	0.055	1.32E−04	0.145	− 1.07E−05	0.043
Nuclear *d*_N_/*d*_S_	− 1.060	− 0.179	−0.030	− 1.72E−04	0.225	4.70E−06	0.050
Nuclear *d*_N_	0.174	0.232	0.042	4.00E−05	− 0.115	− 7.35E−06	0.013
Nuclear *d*_S_	1.655	0.514	0.083	2.79E−04	− 0.464	− 1.58E−05	0.094

*P*-values denoted * ≤ 0.1, ** ≤ 0.05

## Discussion

Our study uncovered associations among wing metrics as proxies of metabolic demands related to flight, environmental temperature, and UV radiation with molecular evolutionary rates across the Neotropical avian family Furnariidae. Our analyses revealed weak evidence for a positive effect of environmental energy on mitochondrial molecular evolutionary rates in these birds, as well as an interaction between temperature and UV radiation in driving molecular evolution (Tables 1, 2). We also found a slight positive effect of body size and wing elongation (measured as hand-wing index) on mitochondrial substitution rates, and a negative interaction between these two variables as drivers of molecular rates (Table 1). Overall, the evidence of the effects of flight demand (as estimated from wing shape) and environmental energy on the genome was stronger on non-synonymous substitution rates (*d*_N_).

A salient observation in our analyses is a strikingly low explanatory power of our models. There are various reasons which may explain such result. First, despite focusing on one of the largest avian families with near-complete sampling, we included a small number of genomic regions. Gene-specific variation in rates, as well as an interaction between the gene-specific and the genome-wide variation among lineages [41] may compound the error (noise) caused by having a finite number of nucleotide sites per gene, thereby resulting in models with limited explanatory power. Another explanation is our use of proxy variables (e.g. wing morphology as an indicator of metabolic demands imposed by flight) to characterize biological attributes of species and our use of summary statistics to simplistically describe the environments occupied by species which may range broadly and encounter a variety of conditions influencing genome evolution across their ranges.

Beyond considerations related to data quality potentially resulting in low effect sizes, there are biological processes which may underlie the patterns we found. Our results are in agreement with previous studies on the positive association between environmental energy and molecular evolutionary rates in birds [11, 42] and on the interaction between temperature and UV radiation as drivers of molecular evolution [29]. To our knowledge, ours is the first evidence presented for an effect, albeit weak, of avian flight demands on molecular rates. We detected an effect only on *d*_N_ substitution rates only through mitochondrial markers for both environmental and morphological variables. The mitochondrial genome evolves faster than the nuclear genome, possibly because of higher DNA damage and less efficient repair mechanisms in mitochondrial DNA [25]. A source of DNA damage is the high exposure to oxidative stress due to the metabolic reactions related to aerobic respiration in mitochondria [43], a potential mechanism by which metabolic rate and UV radiation might affect molecular rates, explaining our finding of a relationship of environmental variables with mitochondrial substitution rates but not with nuclear rates. However, the low number of nuclear sites in this study may have also hampered the detection of associations in the nuclear dataset.

Because mitochondrial *d*_N_ rates reflect amino-acid substitutions, the association of such rates with morphological and environmental variables might occur via selective pressures or through genetic drift mediated by population size, rather than via basal rates of mutation. This result is consistent with previous studies showing that flight has led to a broad range of genomic as well as physiological adaptations [44], and with evidence that metabolic rates do not have a simple association with molecular rates [13]. Given the apparently large amount of noise in our data, we emphasize that genome-wide data and direct measures of metabolic rates will be instrumental in further characterizing the genomic signature of mutation pressures and selection or population size arising from energetically demanding flight and high-energy environments [45].

There are several reasons why metabolic rates might not drive basal mutation rates, as would be expected from theory [7]. Most organisms likely exist at an upper limit of mutations, such that even a minor increase in mutation rate may lead to an unsustainable loss of fitness [1]. For example, large-bodied mammals might accumulate more mutations than small-bodied mammals because the long lifespan of the former allows for more generations of cells producing gametes, and this might interact with the smaller population sizes of larger organisms to result in faster accumulation of deleterious changes [4]. Nonetheless, instead of having a fast rate of accumulation of changes, large-bodied mammals have relatively slow molecular evolutionary rates, possibly reflecting high fitness costs of deleterious changes [46]. Fast metabolic rates in birds with intense flight might pose a similarly increased selective pressure to avoid deleterious mutations and could explain the lack of association between proxies of metabolic rate and those of mutation rates (e.g., [13]).

In agreement with avian aerodynamic theory [47], we found that birds that fly more during their daily activities or their annual cycle tend to have low wing loadings and particularly high aspect ratios (as estimated by the hand-wing index, Fig. 1). Sedentary species arguably experience lower metabolic demands from flight, except for occasional flights of short duration, which can be metabolically demanding due to the comparatively high wing loadings and low aspect ratios in these species. On the other hand, species that fly regularly have higher aspect ratios and can fly more economically but may endure the metabolic demands of flight more frequently and for longer periods. We found evidence for an association between molecular rates with hand-wing index but not with wing loading (Tables 1, 2), suggesting an association of substitution rates with a sustained metabolic demand produced by frequent flight, rather than from potential metabolic peaks produced by rare but costly flights in sedentary species. Our results further suggest a change in the intensity or direction of this effect depending on body mass (Table 1; Fig. 1). This change might be explained by differences in population size or ecology between small and largebodied species; for instance, small species have relatively higher energy requirements for maintenance and thermoregulation, making them more susceptible to the effects of environmental energy and metabolic products [48]. Experimental tests evaluating directly the relationship between metabolic rates and wing morphology would be a fruitful area of future research (e.g., [49]).

We found that temperature and UV radiation may interact to accelerate non-synonymous evolutionary rates in the mitochondrial genome (Fig. 2). In ectotherms, low temperatures have been reported as having a negative impact on DNA repair mechanisms, explaining the interaction of temperature with the mutagenic impact of high exposure to UV radiation [29]. However, our data suggest that environmental energy is associated with molecular evolution through mechanisms other than DNA repair given that we found the association in non-synonymous sites, which are influenced by the interplay between selection and genetic drift [24]. Environments with low temperatures and high UV radiation show also low primary productivity and may impose additional challenges, such as hydric stress in cold Patagonian steppes or reduced oxygen pressure at high altitudes in the Andes. These factors can result in high selective pressures for novel adaptations or reduced population sizes, and these can drive the observed elevated non-synonymous substitution rates. Specifically, adaptations may include efficient mitochondrial respiratory chain complexes optimised for efficient respiration and thermoregulation. Because the density of resources in these environments is low, adaptations for high mobility, including high flight efficiency, are also expected. The furnariid genera *Geositta* and *Cinclodes* are good examples of birds adapted to mobile lifestyles in cold and dry environments [50, 51].

Selection and population size often interact in complex ways to influence substitution rates [33]. For example, the relationship between population size and substitution rate may take drastically different shapes depending on whether a genomic region is under positive or negative selection. In the case of regions largely undergoing negative selection (e.g., many protein-coding loci), large population sizes result in a greater efficiency of selection, leading to relatively low substitution rates even under a constant selection coefficient. This means that our data cannot determine whether wing elongation is associated with substitution rates via an effect on selection or on population size. For instance, our results may reflect a negative association between population size and both flight efficiency and environmental energy. Indeed, adaptations for regular flight such as elongated wings may also be associated with lower population sizes because highly aerial species tend to use resources that are in low density, creating a correlation between flight habits and population density [52, 53]. Incorporating independent estimates of population size might be an alternative to disentangle the effects of selection and population size on molecular evolution in future research.

## Conclusions

Our study establishes an association between wing morphology and flight habits, and provides evidence that flight and environmental energy can impact genome evolution via an influence on natural selection or historical effective population sizes. Our analyses using the hand-wing index suggest that frequent flight is associated with higher substitution rates in mitochondrial genes, a pattern that may result from natural selection or genetic drift. These results support the idea that flight demand is associated with a range of adaptations or effects of population size leading to positive selection on the genome. Meanwhile, our data show further evidence of interacting effects of sources of environmental energy on molecular evolution, but likely tied with a range of adaptations or population constraints found at extreme lifestyles rather than impacting mechanisms of DNA repair. Data from whole genomes and detailed physiology across bird taxa will bring a more complete picture of the impact of metabolism, population size, and the environment on avian genomic evolution.

## Methods

### Data collection

We used data on wing shape and body mass obtained from museum specimens as proxies of the flight demands for 290 species in Furnariidae [54]. Specifically, we measured the distance from the carpal joint to the tip of the longest primary feather (*WL*, the traditional “wing length” measurement) and the distance from the carpal joint to the tip of the first secondary feather (*S1*) and obtained body mass from specimen labels. With these measurements we calculated the hand-wing index as 100(*WL* – *S1*)/*WL* and the wing loading as *M*/3*WLS1*, where *M* is the body mass and 3*WLS1* is an estimate of the total wing area [55]. The hand-wing index is proportional to the aspect ratio of the wings in furnariids [54] and therefore it is related to long-distance flight efficiency [56]. Wing loading is related to the power required for flight regardless of the distance travelled, and is thus associated with the metabolic demand per unit of time in flight [57].

All else being equal, wings with high aspect ratio and low loading are expected to result in lower metabolic demands. However, the efficiency of flight and lifetime metabolic demands may be positively correlated across all birds because species that show highly flight-efficient morphologies also fly more frequently and longer distances [56]. Meanwhile, species with the least efficient flight morphologies tend to avoid flight and thus are expected to experience lower metabolic demands [58]. Therefore, we first examined the relationship between flight efficiency and flight behaviour in Furnariidae to validate the use of flight morphology as a proxy for metabolic demands.

We used basic natural history information to characterize flight behaviour of selected species of Furnariidae. For well-known species in each genus, we collected information from the literature about seasonal movement (0 = year-round sedentary or territorial; 1 = non-breeding seasonal displacements or nomadism; 2 = migratory), foraging behaviour (0 = flight rarely used during foraging, short flights < 10 m seldom needed for crossing habitat gaps or commuting from roosting or nesting sites; 1 = flight used sporadically during foraging, for moving through habitats or for commuting; 2 = flight used regularly for all or nearly all foraging activities), and foraging stratum (0 = ground, low dense vegetation or forest understory; 1 = midstory; 2 = canopy) (Additional file 1: Table S1). We used mostly the information in the species accounts of the Bird of the World website (https://birdsoftheworld.org/bow/home, [59]), with additional or more specific references listed in Table S1. We used the sum of scores divided by the maximum value (6) as an index of the lifelong flight habits of species, and used phylogenetic regression to examine the association between these data and each of hand-wing index and wing loading (see "Regression analyses" section below). Although these data are useful to verify expectations about wing metrics as proxies of metabolic demand, we did not test for the association between flight score and molecular rates due to its restricted sample size.

We collected data on environmental temperature and UV radiation across the ranges of the species in the family Furnariidae. For all species, we downloaded georeferenced records from GBIF (Global Biodiversity Information Facility, https://www.gbif.org/) and VertNet (http://portal.vertnet.org/), discarding duplicated records and those outside the known distribution range for each species according to expert-based maps [60]. Using 145,216 vetted records (mean per species = ~ 615), we estimated the median and standard deviation for the annual mean temperature from WorldClim data [61] at 30 arc-sec resolution, and for the annual mean UV-B from gIUV [62] with 15 arc-min resolution. We choose to focus on median values because these are more robust to atypical values. Because median and mean values show nearly identical results, the results obtained using mean values are shown only in supplementary material (Additional file 1: Tables S2, S3). Among the environmental variables available, temperature and UV provide a broad description of environmental energy, yet they are not simply correlated to each other; these variables follow a triangular-shaped association, where high values of UV occur both in habitats with high and low temperatures (e.g., high altitudes). Because current geographic distributions may differ greatly from distributions in the past, contemporary measurements of environmental variables can introduce noise to subsequent phylogenetic regression analyses. This noise is likely to increase the Type II error rate but it is unlikely to cause a bias that increases Type I error rate, so analyses of this type of data sets are in fact conservative [28].

Genomic data were taken from a published phylogenetic study of Furnariidae analysing sequence data from mitochondrial and nuclear markers collected for nearly every species in the family [36]. We used two nuclear and three mitochondrial loci (4023 nt of *recombination activating genes 1* and *2*; and 2076 nt of *NADH dehydrogenase subunits 2* and *3*, and *cytochrome oxidase subunit 2*, respectively) from the source phylogenetic study of Furnariidae. We selected species included in the phylogeny for which data for environmental variables, wing loading, and hand-wing index were available. Our analyses focused exclusively on species with measurements of wing metrics from known museum specimens, and species for which molecular rates could be estimated with confidence (non-zero values, see below; *N* nuclear data = 55; *N* mitochondrial data = 184; Additional file 1: Fig. S3). A nuclear intron used in the original study was not included. The molecular data and variables used in subsequent regression analyses are available online (github.com/duchene/furnariidae_rates).

### Estimates of rates of molecular evolution

Absolute rates of expected synonymous and non-synonymous substitutions per nucleotide site per million years were estimated form the sequence alignments along the branches of an existing time-calibrated tree of Furnariidae [36]. To make reliable analyses of molecular evolutionary rates, we first tested for potential biases from the molecular substitution model using simulation-based tests of model adequacy and substitution saturation as implemented in the software PhyloMAd [63]. This procedure assesses whether empirical data adhere to the assumptions made by the model by comparing them with data simulated under the model. We then estimated the expected number of synonymous and non-synonymous substitutions along branches (expressed as branch lengths) using the MG94 model of codon evolution with codon frequencies estimated from the data [64], implemented in HyPhy v2.5 [65]. This model was used in combination with the best-fitting nucleotide substitution model of the GTR + Γ family [66]. Synonymous and non-synonymous substitutions along branches were estimated independently for mitochondrial and nuclear data. Terminal molecular branch-length estimates were divided by the published time-estimate for each branch [36] and taken as the mean molecular evolutionary rates of species branches.

The resulting synonymous substitution rates (*d*_S_) are the rates of molecular changes not influencing the amino acid coding, and are proportional to the mutation rate under the condition of no bias in codon usage [67]. Meanwhile, non-synonymous substitution rates (*d*_N_) represent rates of amino-acid substitutions, and thus reflect the mutation rate and the interaction between selection and population size [24]. The *d*_N_/*d*_S_ ratio is therefore expected to reflect the effects from selection and population size, excluding the effect of the mutation rate.

### Regression analyses

We used phylogenetic generalized least-squares (PGLS) regression models to examine the relationship between wing morphology, environmental energy, and molecular evolutionary rates. Hypotheses were tested independently using nuclear and mitochondrial data, and using each of *d*_N_, *d*_S_ and *d*_N_/*d*_S_ rates as response variables. Explanatory variables in the models included the hand-wing index or wing loading, median environmental temperature, and median UV radiation. We included body mass as explanatory variable because of its known effects on metabolic rates [7] and because it affects most life-history traits (e.g., generation time, lifespan) and demography, so it has a possible confounding effect of the association between wing morphology variables, environmental variables, and molecular evolution [4]. Finally, we also incorporated the interaction terms between the explanatory variables to consider differences in biology between species of different body sizes, and the interplay between the environmental temperature and UV radiation (*d*_*N*_ *|d*_*S*_ *|d*_*N*_*/d*_*S*_ ~ hand-wing index*log(BodyMass) + Temp*UV radiation or *d*_*N*_ *|d*_*S*_ *|d*_*N*_*/d*_*S*_ ~ wing loading*log(BodyMass) + Temp*UV radiation). Given the different nature and units of each variable, we used standardized values (mean = 0, standard deviation = 1) for all variables included in the models.

To account for non-independence of data due to relatedness among taxa, we used a species-level phylogeny from the original phylogenetic study [36]. We normalized the molecular variables using the normalize() function of the R package *BBmisc* [68], and then performed a Box-Cox transformation on all molecular variables and log transformation on body mass to adhere to least-squares model assumptions. The lambda parameter for the Box-Cox transformation was estimated with boxcox() function of the *MASS* R package [69], adding as a constant value the minimum in each variable plus 0.01 on the respective variable, to avoid negative values. PGLS regression was performed under a lambda model of trait evolution, such that we estimated the best fitting value of phylogenetic inertia in the residuals simultaneously with other parameters [70]. The parameters of PGLS regression models were optimized using the pgls() function of the *caper* R package [71], and residuals for each model were assessed visually for normality. Because regression models containing each of the molecular variables and environmental variables were ran twice, we verified that any results remained qualitatively identical by adjusting *p*-values using false discovery rates. Tendency lines for Figs. 1 and 2, were generated through predict.plgs() function from *caper* package. Figures were generated using *ggplot2* package [72] for R.

The small genomic sample size in our data means that regression analyses are unlikely to be biased towards Type I errors, but are primarily be prone to Type II errors. We examined the robustness of any significant regression terms as well as the chances of a Type II error by performing a randomised test of sensitivity to taxon sampling. One thousand replicate regression analyses were performed, each excluding a randomly-selected 25% of the taxa in the complete data set. Results of slope and *t*-statistic were taken to be worthy of mention if they did not overlap zero across randomisations.

## Supplementary information

**Additional file 1: Fig. S1**. Distribution of *t*-statistics for variables including across 1000 replicate regression analyses, each excluding a random set of 25% of species. The model evaluated the explanatory power of wing loading, body mass, median annual temperature and UV radiation on mitochondrial and nuclear molecular rates. Overlap between* t* values and 0 indicates non-significant relationships. **Fig. S2**. Distribution of *t*-statistics for variables including across 1000 replicate regression analyses, each excluding a random set of 25% of species. The model evaluated the explanatory power of hand-wing index, body mass, median annual temperature and UV radiation on mitochondrial and nuclear molecular rates. Overlap between *t* values and 0 indicates non-significantrelation ships. **Fig. S3**. Phylogenetic estimate of the family Furnariidae [36], showing the taxa included in regression models with mitochondrial (red) and nuclear (orange) data. Inclusion depended on availability of data on all of the variables describing environment, wing loading, hand-wing index, and molecular evolutionary rates. **Table S1**. Flight habit scores for 45 species in the Furnariidae. Scores are based on seasonal movement, foraging behaviour, and foraging stratum according to natural history data published. Higher score, more intensive is the flight habit. **Table S2**. PGLS regression coefficients from models testing whether the hand-wing index and mean values for environmental variables explain molecular rates. A Box-Cox transformation was used for all molecular rates variables. **Table S3**. PGLS regression coefficients from models testing whether the wing loading index and mean values for environmental variables explain molecular rates. A Box-Cox transformation was used for all molecular rates variables.

## Data Availability

The molecular sequence alignments and data used for regression analyses in this project are freely available at github.com/duchene/furnariidae_rates.
