# Supplementary material for: Environmental niche and flight intensity are associated with molecular evolutionary rates in a large avian radiation
Source: BMC Ecol Evol. 2022 Aug 2;22:95. doi: 10.1186/s12862-022-02047-0 (PMC9347078; doi:10.1186/s12862-022-02047-0)
Supplement: Supplementary file 1 — Additional file 1: Fig. S1. Distribution of t-statistics for variables including across 1000 replicate regression analyses, each excluding a random set of 25% of species. The model evaluated the explanatory power of wing loading, body mass, median annual temperature and UV radiation on mitochondrial and nuclear molecular rates. Overlap between t values and 0 indicates non-significant relationships. Fig. S2. Distribution of t-statistics for variables including across 1000 replicate regression analyses, each excluding a random set of 25% of species. The model evaluated the explanatory power of hand-wing index, body mass, median annual temperature and UV radiation on mitochondrial and nuclear molecular rates. Overlap between t values and 0 indicates non-significantrelation ships. Fig. S3. Phylogenetic estimate of the family Furnariidae [36], showing the taxa included in regression models with mitochondrial (red) and nuclear (orange) data. Inclusion depended on availability of data on all of the variables describing environment, wing loading, hand-wing index, and molecular evolutionary rates. Table S1. Flight habit scores for 45 species in the Furnariidae. Scores are based on seasonal movement, foraging behaviour, and foraging stratum according to natural history data published. Higher score, more intensive is the flight habit. Table S2. PGLS regression coefficients from models testing whether the hand-wing index and mean values for environmental variables explain molecular rates. A Box-Cox transformation was used for all molecular rates variables. Table S3. PGLS regression coefficients from models testing whether the wing loading index and mean values for environmental variables explain molecular rates. A Box-Cox transformation was used for all molecular rates variables. [file 12862_2022_2047_MOESM1_ESM.docx]

**Supporting information**

**Environmental niche and flight intensity are associated with molecular evolutionary rates in a large avian radiation**

Paola Montoya^a,b,1^, Carlos Daniel Cadena^b^, Santiago Claramunt^c,d^, David Alejandro Duchêne^e^

^a^Instituto de Investigación de Recursos Biológicos Alexander von Humboldt, Bogotá, Colombia

^b^Departamento de Ciencias Biológicas, Universidad de los Andes, Apartado 4976, Bogotá, Colombia

^c^Department of Natural History, Royal Ontario Museum, 100 Queen’s Park Crescent, Toronto, Ontario M5S 2C6, Canada

^d^Department of Ecology and Evolutionary Biology, University of Toronto, Toronto, Ontario M5S 1A1, Canada

^e^Centre for Evolutionary Hologenomics, University of Copenhagen, Øster

Farimagsgade 5A, 1352 Copenhagen, Denmark

^1^Corresponding author

Paola Montoya

Instituto de Investigación de Recursos Biológicos Alexander von Humboldt

Avenida Circunvalar # 16 - 20

Bogotá, Colombia

Telephone: +57 1 320 27 67

Email: pa.montoya18@gmail.com

**Figure S1**. Distribution of t-statistics for variables including across 1000 replicate regression analyses, each excluding a random set of 25% of species. The model evaluated the explanatory power of wing loading, body mass, median annual temperature and UV radiation on mitochondrial and nuclear molecular rates. Overlap between *t* values and 0 indicates non-significant relationships.

**Figure S2**. Distribution of t-statistics for variables including across 1000 replicate regression analyses, each excluding a random set of 25% of species. The model evaluated the explanatory power of hand-wing index, body mass, median annual temperature and UV radiation on mitochondrial and nuclear molecular rates. Overlap between *t* values and 0 indicates non-significant relationships.

**Figure S3**. Phylogenetic estimate of the family Furnariidae [36], showing the taxa included in regression models with mitochondrial (red) and nuclear (orange) data. Inclusion depended on availability of data on all of the variables describing environment, wing loading, hand-wing index, and molecular evolutionary rates.

**
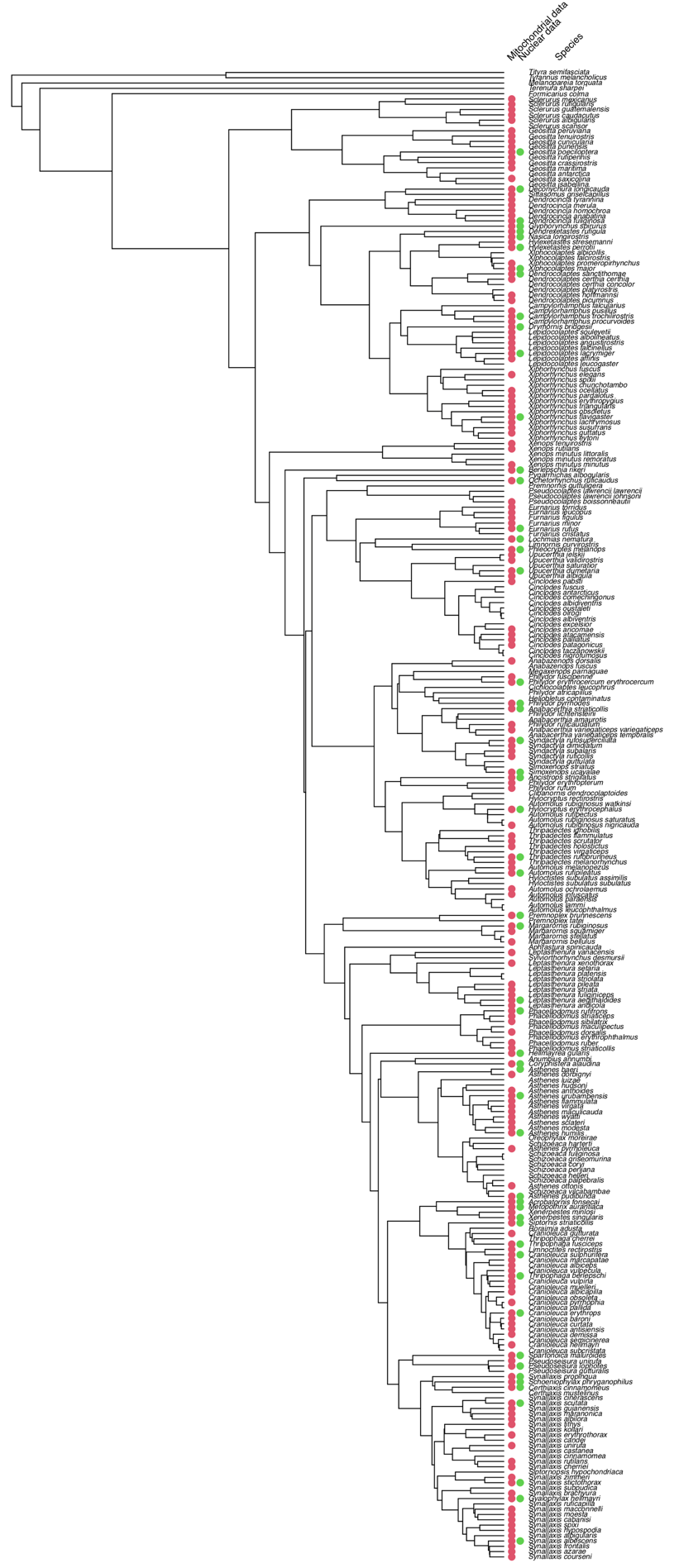
**

**Table S1**. Flight habit scores for 45 species in the Furnariidae. Scores are based on seasonal movement, foraging behaviour, and foraging stratum according to natural history data published. Higher score, more intensive is the flight habit.

| **Species** | **Score** | **References** |
| --- | --- | --- |
| *Acrobatornis fonsecai* | 0.67 | Pacheco, J., Whitney, B., Conzaga, L., (1996). A new genus and species of furnariid from the cocoa-growing region of southeastern Bahia, Brazil. Wilson Bulletin 108: 397-606 |
| *Anabacerthia amaurotis* | 0.50 | Parrini, R. & Pacheco, J. F. 2011. Observações adicionais sobre o comportamento de forrageamento de *Anabacerthia amaurotis* (Passeriformes: Furnariidae) na Mata Atlântica do sudeste do Brasil Atualidades Ornitológicas 160: 33-37.  Remsen, Jr., J. V. (2020). White-browed Foliage-gleaner (*Anabacerthia amaurotis*), version 1.0. In Birds of the World (J. del Hoyo, A. Elliott, J. Sargatal, D. A. Christie, and E. de Juana, Editors). Cornell Lab of Ornithology, Ithaca, NY, USA. https://doi-org.myaccess.library.utoronto.ca/10.2173/bow.whbfog1.01 |
| *Anabazenops dorsalis* | 0.17 | Kratter, A.W. and Parker, T.A. (1997). Relationship of two bamboo-specialized foliage-gleaners: Automolus dorsalis and Anabazenops fuscus (Furnariidae). Pp. 383–397 in: Remsen (1997). |
| *Ancistrops strigilatus* | 0.58 | Remsen, Jr., J. V. (2020). Chestnut-winged Hookbill (Ancistrops strigilatus), version 1.0. In Birds of the World (J. del Hoyo, A. Elliott, J. Sargatal, D. A. Christie, and E. de Juana, Editors). Cornell Lab of Ornithology, Ithaca, NY, USA. https://doi-org.myaccess.library.utoronto.ca/10.2173/bow.chwhoo1.01 |
| *Anumbius annumbi* | 0.33 | Hudson, W. H. (1920). Birds of La Plata, vol. 1. Dent & Sons, London. |
| *Aphrastura spinicauda* | 0.67 | Botero‐Delgadillo, E., Quirici, V., Poblete, Y., Poulin, E., Kempenaers, B. and Vásquez, R.A., 2020. Exploratory behavior, but not aggressiveness, is correlated with breeding dispersal propensity in the highly philopatric thorn‐tailed rayadito. Journal of Avian Biology, 51(2). |
| *Asthenes luizae* | 0.00 | Freitas, G.H.S., Costa, L.M. and Rodrigues, M. (2019), Home‐ranges, population densities, vocal behavior, and post‐fledging movements of Cipo Canasteros (*Asthenes luizae*, Furnariidae), a rock‐specialist endemic of the highlands of eastern Brazil. J. Field Ornithol., 90: 215-228. doi:10.1111/jofo.12308 |
| *Automolus ochrolaemus* | 0.17 | Skutch, Alexander F. (1969). Life Histories of Central American Birds III: Families Cotingidae, Pipridae, Formicariidae, Furnariidae, Dendrocolaptidae, and Picidae. Cooper Ornithological Society, Berkeley, California. Remsen, Jr., J. V. and H. F. Greeney (2020). Buff-throated Foliage-gleaner (*Automolus ochrolaemus*), version 1.0. In Birds of the World (S. M. Billerman, B. K. Keeney, P. G. Rodewald, and T. S. Schulenberg, Editors). Cornell Lab of Ornithology, Ithaca, NY, USA. https://doi-org.myaccess.library.utoronto.ca/10.2173/bow.btfgle1.01 |
| *Berlepschia rikeri* | 0.67 | Esteves Lopes, L. & I. P. de Faria (2014) Range and natural history of point-tailed palmcreepers (Aves: Furnariidae), Journal of Natural History, 48:19-20, 1217-1224, DOI: 10.1080/00222933.2013.862313 |
| *Campylorhamphus trochilirostris* | 0.33 | Marantz, C. A., A. Aleixo, L. R. Bevier, and M. A. Patten (2020). Red-billed Scythebill (Campylorhamphus trochilirostris), version 1.0. In Birds of the World (J. del Hoyo, A. Elliott, J. Sargatal, D. A. Christie, and E. de Juana, Editors). Cornell Lab of Ornithology, Ithaca, NY, USA. https://doi-org.myaccess.library.utoronto.ca/10.2173/bow.rebscy1.01 |
| *Cinclodes fuscus* | 0.67 | Remsen, Jr., J. V. and G. M. Kirwan (2020). Buff-winged Cinclodes (Cinclodes fuscus), version 1.0. In Birds of the World (J. del Hoyo, A. Elliott, J. Sargatal, D. A. Christie, and E. de Juana, Editors). Cornell Lab of Ornithology, Ithaca, NY, USA. https://doi-org.myaccess.library.utoronto.ca/10.2173/bow.buwcin1.01 |
| *Cranioleuca pyrrhophia* | 0.58 | Blendinger, P. G. (2005). Foraging behaviour of birds in an arid sand-dune scrubland in Argentina. Emu-Austral Ornithology, 105(1), 67-79. |
| *Deconychura longicauda* | 0.58 | Marantz, C. A., J. del Hoyo, N. Collar, A. Aleixo, L. R. Bevier, G. M. Kirwan, and M. A. Patten (2020). Long-tailed Woodcreeper (*Deconychura longicauda*), version 1.0. In Birds of the World (S. M. Billerman, B. K. Keeney, P. G. Rodewald, and T. S. Schulenberg, Editors). Cornell Lab of Ornithology, Ithaca, NY, USA. https://doi-org.myaccess.library.utoronto.ca/10.2173/bow.lotwoo1.01 |
| *Dendrexetastes rufigula* | 0.42 | Marantz, C. A., A. Aleixo, L. R. Bevier, and M. A. Patten (2020). Cinnamon-throated Woodcreeper (Dendrexetastes rufigula), version 1.0. In Birds of the World (J. del Hoyo, A. Elliott, J. Sargatal, D. A. Christie, and E. de Juana, Editors). Cornell Lab of Ornithology, Ithaca, NY, USA. https://doi-org.myaccess.library.utoronto.ca/10.2173/bow.citwoo1.01 |
| *Dendrocincla fuliginosa* | 0.50 | Chapman, A., & Rosenberg, K. V. (1991). Diets of four sympatric Amazonian woodcreepers (Dendrocolaptidae). The Condor, 93(4), 904-915. Marantz, C. A., A. Aleixo, L. R. Bevier, M. A. Patten, and H. F. Greeney (2020). Plain-brown Woodcreeper (Dendrocincla fuliginosa), version 1.0. In Birds of the World (S. M. Billerman, B. K. Keeney, P. G. Rodewald, and T. S. Schulenberg, Editors). Cornell Lab of Ornithology, Ithaca, NY, USA. https://doi-org.myaccess.library.utoronto.ca/10.2173/bow.plbwoo1.01 |
| *Dendroplex picus* | 0.33 | Marantz, C. A., A. Aleixo, L. R. Bevier, M. A. Patten, and E. de Juana (2020). Straight-billed Woodcreeper (Dendroplex picus), version 1.0. In Birds of the World (J. del Hoyo, A. Elliott, J. Sargatal, D. A. Christie, and E. de Juana, Editors). Cornell Lab of Ornithology, Ithaca, NY, USA. https://doi-org.myaccess.library.utoronto.ca/10.2173/bow.stbwoo2.01 |
| *Drymornis bridgesii* | 0.17 | Kratter, A. W., Sillett, T. S., Chesser, R. T., O'Neill, J. P., Parker III, T. A., & Castillo, A. (1993). Avifauna of a Chaco locality in Bolivia. The Wilson Bulletin, 114-141.  Juárez-Jovel, R. C. (2021). Scimitar-billed Woodcreeper (Drymornis bridgesii), version 2.0. In Birds of the World (S. M. Billerman, Editor). Cornell Lab of Ornithology, Ithaca, NY, USA. https://doi-org.myaccess.library.utoronto.ca/10.2173/bow.scbwoo4.02 |
| *Furnarius rufus* | 0.17 | Hudson, W. H. (1920). Birds of La Plata, vol. 1. Dent & Sons, London.  Remsen, Jr., J. V. and A. Bonan (2020). Rufous Hornero (Furnarius rufus), version 1.0. In Birds of the World (J. del Hoyo, A. Elliott, J. Sargatal, D. A. Christie, and E. de Juana, Editors). Cornell Lab of Ornithology, Ithaca, NY, USA. https://doi-org.myaccess.library.utoronto.ca/10.2173/bow.rufhor2.01 |
| *Geositta cunicularia* | 0.50 | Hudson, W. H. (1920). Birds of La Plata, vol. 1. Dent & Sons, London. |
| *Glyphorynchus spirurus* | 0.50 | Skutch, A.F. (1969). Life Histories of Central American Birds. III. Pacific Coast Avifauna 35. Cooper Ornithological Society, Berkeley, California. 580 pp. Marantz, C. A., A. Aleixo, L. R. Bevier, M. A. Patten, and D. A. Christie (2020). Wedge-billed Woodcreeper (Glyphorynchus spirurus), version 1.0. In Birds of the World (J. del Hoyo, A. Elliott, J. Sargatal, D. A. Christie, and E. de Juana, Editors). Cornell Lab of Ornithology, Ithaca, NY, USA. https://doi-org.myaccess.library.utoronto.ca/10.2173/bow.webwoo1.01 |
| *Hylocryptus rectirostris* | 0.00 | Faria, L.C.P., Carrara, L.A. and Rodrigues, M., 2007. Sistema territorial e forrageamento do fura-barreira *Hylocryptus rectirostris* (Aves: Furnariidae). Revista Brasileira de Ornitologia, 15(3), pp.395-402. |
| *Lepidocolaptes angustirostris* | 0.33 | Blendinger, P. G. (2005). Foraging behaviour of birds in an arid sand-dune scrubland in Argentina. Emu-Austral Ornithology, 105(1), 67-79. |
| *Leptasthenura platensis* | 0.33 | Blendinger, P. G. (2005). Foraging behaviour of birds in an arid sand-dune scrubland in Argentina. Emu-Austral Ornithology, 105(1), 67-79. |
| *Limnoctites rectirostris* | 0.00 | López-Lanús, B., Di Giacomo, A. G., & Babarskas, M. (1999). Estudios sobre ecologia y comportamiento de la Pajonalera Pico Recto Limnoctites rectirostris en la Reserva Otamendi, Buenos Aires, Argentina. Cotinga, 12, 61-63. |
| *Lochmias nematura* | 0.00 | Remsen, Jr., J. V., A. Bonan, and E. de Juana (2020). Sharp-tailed Streamcreeper (Lochmias nematura), version 1.0. In Birds of the World (J. del Hoyo, A. Elliott, J. Sargatal, D. A. Christie, and E. de Juana, Editors). Cornell Lab of Ornithology, Ithaca, NY, USA. https://doi-org.myaccess.library.utoronto.ca/10.2173/bow.shtstr1.01 |
| *Margarornis squamiger* | 0.33 | Remsen, Jr., J. V. (2020). Pearled Treerunner (Margarornis squamiger), version 1.0. In Birds of the World (J. del Hoyo, A. Elliott, J. Sargatal, D. A. Christie, and E. de Juana, Editors). Cornell Lab of Ornithology, Ithaca, NY, USA. <https://doi-org.myaccess.library.utoronto.ca/10.2173/bow.peatre1.01> |
| *Phacellodomus rufifrons* | 0.00 | Thomas, B.T., 1983. The plain-fronted thornbird: nest construction, material choice, and nest defense behavior. Wilson Bulletin 106-117. |
| *Philydor erythrocercum* | 0.58 | Remsen, Jr., J. V. (2020). Rufous-rumped Foliage-gleaner (Philydor erythrocercum), version 1.0. In Birds of the World (J. del Hoyo, A. Elliott, J. Sargatal, D. A. Christie, and E. de Juana, Editors). Cornell Lab of Ornithology, Ithaca, NY, USA. <https://doi-org.myaccess.library.utoronto.ca/10.2173/bow.rurfog1.01> |
| *Phleocryptes melanops* | 0.33 | Remsen, Jr., J. V., E. de Juana, and G. M. Kirwan (2020). Wren-like Rushbird (Phleocryptes melanops), version 1.0. In Birds of the World (J. del Hoyo, A. Elliott, J. Sargatal, D. A. Christie, and E. de Juana, Editors). Cornell Lab of Ornithology, Ithaca, NY, USA. <https://doi-org.myaccess.library.utoronto.ca/10.2173/bow.wrlrus1.01> |
| *Premnoplex brunnescens* | 0.25 | Areta, I.J. (2007). Behavior and phylogenetic position of *Premnoplex* barbtails (Furnariidae). Condor 109(2), 399-407. |
| *Pseudasthenes cactorum* | 0.00 | Remsen, Jr., J. V. and E. de Juana (2020). Cactus Canastero (Pseudasthenes cactorum), version 1.0. In Birds of the World (J. del Hoyo, A. Elliott, J. Sargatal, D. A. Christie, and E. de Juana, Editors). Cornell Lab of Ornithology, Ithaca, NY, USA. https://doi-org.myaccess.library.utoronto.ca/10.2173/bow.caccan1.01 |
| *Pseudocolaptes boissonneautii* | 0.58 | Remsen, Jr., J. V. (2020). Streaked Tuftedcheek (Pseudocolaptes boissonneautii), version 1.0. In Birds of the World (J. del Hoyo, A. Elliott, J. Sargatal, D. A. Christie, and E. de Juana, Editors). Cornell Lab of Ornithology, Ithaca, NY, USA. <https://doi-org.myaccess.library.utoronto.ca/10.2173/bow.strtuf1.01> |
| *Pseudoseisura lophotes* | 0.17 | Kratter, A. W., Sillett, T. S., Chesser, R. T., O'Neill, J. P., Parker III, T. A., & Castillo, A. (1993). Avifauna of a Chaco locality in Bolivia. The Wilson Bulletin, 114-141.  Blendinger, P. G. (2005). Foraging behaviour of birds in an arid sand-dune scrubland in Argentina. Emu-Austral Ornithology, 105(1), 67-79. |
| *Asthenes griseomurina* | 0.00 | Schulenberg, T. S. and T. Johnson (2020). Mouse-colored Thistletail (Asthenes griseomurina), version 1.0. In Birds of the World (T. S. Schulenberg, Editor). Cornell Lab of Ornithology, Ithaca, NY, USA. https://doi-org.myaccess.library.utoronto.ca/10.2173/bow.mocthi1.01 |
| *Schoeniophylax phryganophilus* | 0.00 | Remsen, Jr., J. V. (2020). Chotoy Spinetail (Schoeniophylax phryganophilus), version 1.0. In Birds of the World (J. del Hoyo, A. Elliott, J. Sargatal, D. A. Christie, and E. de Juana, Editors). Cornell Lab of Ornithology, Ithaca, NY, USA. https://doi-org.myaccess.library.utoronto.ca/10.2173/bow.chospi2.01 |
| *Sclerurus mexicanus* | 0.17 | Cooper, J. C., D. F. Barragán, R. C. Juárez-Jovel, and P. F. D. Boesman (2021). Middle American Leaftosser (Sclerurus mexicanus), version 1.1. In Birds of the World (T. S. Schulenberg, Editor). Cornell Lab of Ornithology, Ithaca, NY, USA. https://doi-org.myaccess.library.utoronto.ca/10.2173/bow.tatlea1.01.1 |
| *Sittasomus griseicapillus* | 0.50 | Parrini, R. & Pacheco, J. F. 2011. Comportamento de forrageamento do arapaçu-verde *Sittasomus griseicapillus* (Passeriformes: Dendrocolaptidae) na Mata Atlântica do Estado do Rio de Janeiro, sudeste do Brasil. Atualidades Ornitológicas 161: 33-39. Patten, M. A. (2020). Olivaceous Woodcreeper (Sittasomus griseicapillus), version 1.0. In Birds of the World (T. S. Schulenberg, Editor). Cornell Lab of Ornithology, Ithaca, NY, USA. https://doi-org.myaccess.library.utoronto.ca/10.2173/bow.oliwoo1.01 |
| *Spartonoica maluroides* | 0.17 | Llambías, P. E. (2020). Bay-capped Wren-Spinetail (Spartonoica maluroides), version 1.0. In Birds of the World (T. S. Schulenberg, Editor). Cornell Lab of Ornithology, Ithaca, NY, USA. https://doi-org.myaccess.library.utoronto.ca/10.2173/bow.bcwspi1.01 |
| *Synallaxis azarae* | 0.00 | Remsen, Jr., J. V. (2020). Azara's Spinetail (Synallaxis azarae), version 1.0. In Birds of the World (J. del Hoyo, A. Elliott, J. Sargatal, D. A. Christie, and E. de Juana, Editors). Cornell Lab of Ornithology, Ithaca, NY, USA. https://doi-org.myaccess.library.utoronto.ca/10.2173/bow.azaspi1.01 |
| *Thripadectes rufobrunneus* | 0.00 | Skutch, A.F. (1969). Life Histories of Central American Birds. III. Pacific Coast Avifauna 35. Cooper Ornithological Society, Berkeley, California. 580 pp. |
| *Upucerthia albigula* | 0.17 | Schulenberg, T. S. (2020). White-throated Earthcreeper (Upucerthia albigula), version 1.0. In Birds of the World (T. S. Schulenberg, Editor). Cornell Lab of Ornithology, Ithaca, NY, USA. https://doi-org.myaccess.library.utoronto.ca/10.2173/bow.whtear1.01 |
| *Xenerpestes singularis* | 0.67 | Parker, T.A. and Parker, S.A. (1980). Rediscovery of Xenerpestes singularis (Furnariidae). Auk. 97(1): 203-205. |
| *Xenops rutilans* | 0.50 | Parrini, R. & Pacheco, J. F. 2011. Aspectos do comportamento de forrageamento de *Xenops rutilans* (Passeriformes: Furnariidae) na Mata Atlântica do sudeste do Brasil Atualidades Ornitológicas 164: 33-36. Remsen, Jr., J. V. (2020). Streaked Xenops (Xenops rutilans), version 1.0. In Birds of the World (J. del Hoyo, A. Elliott, J. Sargatal, D. A. Christie, and E. de Juana, Editors). Cornell Lab of Ornithology, Ithaca, NY, USA. <https://doi-org.myaccess.library.utoronto.ca/10.2173/bow.strxen1.01> |
| *Xiphocolaptes promeropirhynchus* | 0.33 | Smith, T. (2020). Strong-billed Woodcreeper (Xiphocolaptes promeropirhynchus), version 1.0. In Birds of the World (T. S. Schulenberg, Editor). Cornell Lab of Ornithology, Ithaca, NY, USA. https://doi-org.myaccess.library.utoronto.ca/10.2173/bow.stbwoo1.01 |
| *Xiphorhynchus guttatus* | 0.50 | Chapman, A., & Rosenberg, K. V. (1991). Diets of four sympatric Amazonian woodcreepers (Dendrocolaptidae). The Condor, 93(4), 904-915. |

**Table S2.** PGLS regression coefficients from models testing whether the hand-wing index and mean values for environmental variables explain molecular rates. A Box-Cox transformation was used for all molecular rates variables.

| **Molecular rate response regression terms** | **Hand-wing index** | **Log body mass** | **Mean annual temperature** | **Mean UV radiation** | **Hand-wing index * log body mass** | **Mean annual temperature * mean UV radiation** | ***R*^2^** |
| --- | --- | --- | --- | --- | --- | --- | --- |
| **Mitochondrial *d*_N_/*d*_S_** | 0.089 * | 0.436 * | 0.046 | 1.17E-04 | -0.025 * | -8.63E-06 | 0.022 |
| **Mitochondrial *d*_N_** | 0.112 * | 0.662 ** | 0.083 * | 2.04E-04 ** | -0.034 * | -1.60E-05 ** | 0.045 |
| **Mitochondrial *d*_S_** | 0.090 | 0.610 * | 0.063 | 1.45E-04 | -0.025 | -1.14E-05 | 0.030 |
| **Nuclear *d*_N_/*d*_S_** | 0.080 | 0.474 | -0.063 | -2.20E-04 | -0.020 | 1.09E-05 | 0.045 |
| **Nuclear *d*_N_** | 0.048 | 0.061 | 0.033 | 9.70E-05 | -0.008 | -5.32E-06 | 0.018 |
| **Nuclear *d*_S_** | -0.036 | -0.626 | 0.127 | 4.35E-04 | 0.016 | -2.32E-05 | 0.092 |

*P*-values denoted * ≤ 0.1, ** ≤ 0.05

**Table S3.** PGLS regression coefficients from models testing whether the wing loading index and mean values for environmental variables explain molecular rates. A Box-Cox transformation was used for all molecular rates variables.

| **Molecular rate response regression terms** | **Wing loading** | **Log body mass** | **Mean annual temperature** | **Mean UV radiation** | **Wing loading * log body mass** | **Mean annual temperature * mean UV radiation** | ***R*^2^** |
| --- | --- | --- | --- | --- | --- | --- | --- |
| **Mitochondrial *d*_N_/*d*_S_** | 0.314 | 0.274 | 0.048 | 1.11E-04 | -0.117 | -8.90E-06 | 0.015 |
| **Mitochondrial *d*_N_** | 0.137 | 0.087 | 0.089 * | 2.12E-04 * | -0.012 | -1.67E-05 * | 0.032 |
| **Mitochondrial *d*_S_** | -0.251 | -0.259 | 0.058 | 1.46E-04 | 0.148 | -1.10E-05 | 0.042 |
| **Nuclear *d*_N_/*d*_S_** | -0.998 | -0.153 | -0.053 | -2.32E-04 | 0.211 | 9.35E-06 | 0.049 |
| **Nuclear *d*_N_** | 0.212 | 0.231 | 0.004 | -1.20E-05 | -0.117 | 1.81E-07 | 0.009 |
| **Nuclear *d*_S_** | 1.646 | 0.491 | 0.065 | 2.75E-04 | -0.456 | -1.21E-05 | 0.099 |

*P*-values denoted * ≤ 0.1, ** ≤ 0.05
